# Impact and Influence of the Natural *Vibrio*-Squid Symbiosis in Understanding Bacterial–Animal Interactions

**DOI:** 10.3389/fmicb.2016.01982

**Published:** 2016-12-15

**Authors:** Mark J. Mandel, Anne K. Dunn

**Affiliations:** ^1^Department of Microbiology-Immunology, Northwestern University Feinberg School of MedicineChicago, IL, USA; ^2^Department of Microbiology and Plant Biology, University of OklahomaNorman, OK, USA

**Keywords:** symbiosis, microbiome, invertebrate model, marine microbiology, epithelial colonization, evolution

## Abstract

Animals are colonized by bacteria, and in many cases partners have co-evolved to perform mutually beneficial functions. An exciting and ongoing legacy of the past decade has been an expansion of technology to enable study of natural associations *in situ*/*in vivo*. As a result, more symbioses are being examined, and additional details are being revealed for well-studied systems with a focus on the interactions between partners in the native context. With this framing, we review recent literature from the *Vibrio fischeri*–*Euprymna scolopes* symbiosis and focus on key studies that have had an impact on understanding bacteria–animal interactions broadly. This is not intended to be a comprehensive review of the system, but rather to focus on particular studies that have excelled at moving from pattern to process in facilitating an understanding of the molecular basis to intriguing observations in the field of host–microbe interactions. In this review we discuss the following topics: processes regulating strain and species specificity; bacterial signaling to host morphogenesis; multiple roles for nitric oxide; flagellar motility and chemotaxis; and efforts to understand unannotated and poorly annotated genes. Overall these studies demonstrate how functional approaches *in vivo* in a tractable system have provided valuable insight into general principles of microbe–host interactions.

## Introduction

Studies of human, animal, and plant microbiomes have been advanced by novel culture-independent approaches and technological advancements in DNA sequencing. In recent years a prominent role for microbial communities of the gut, skin, and other organs has emerged as modulators of human health (Human Microbiome Project Consortium, 2012). These studies followed from influential animal studies in systems that are yielding critical insight into microbiome assembly, stability, communication, and evolution (Ruby, 2008; McFall-Ngai et al., 2013). The focus of this review is to examine one model system, the *Vibrio fischeri*–*Euprymna scolopes* symbiosis, and how key findings in that system have enabled an increasingly higher resolution of the processes and principles that underlie microbe–host communication.

When Hawaiian bobtail squid hatch from their eggs, they are exposed to a million bacteria in each milliliter of seawater. Although *V. fischeri* make up less than 1 in 5,000 of these planktonic, environmental bacteria, the “light organ” of the hatchling squid becomes colonized exclusively with *V. fischeri* (Ruby and Lee, 1998; Mandel, 2010). The microbe–host specificity relies on a series of reciprocal communications between the partners, many of which are detailed in the sections below. Over the course of 48 h the bacteria establish a mature colonization in epithelium-lined crypts of the squid light organ, and, at high cell density, produce light as a result of quorum-sensing. The bacterial bioluminescence is reflected by host tissue to camouflage the shadow or silhouette that the nocturnal-foraging squid would cast in the moonlight, thus protecting the host in a process termed counter-illumination (Ruby and McFall-Ngai, 1992; Jones and Nishiguchi, 2004). Initiation of colonization occurs in newly hatched squid, seeding an individual host’s crypts for its lifetime. The bacteria produce light at night, then at dawn approximately 90–95% of the symbiotic population is expelled into the seawater (Lee and Ruby, 1994; Boettcher et al., 1996; Nyholm and McFall-Ngai, 1998). The remaining cells grow up during the day, produce light at night, and a diel cycle of growth, light production, and expulsion proceeds for the lifetime of the animal (Wier et al., 2010). Host cellular changes accompany this cycle, e.g., a daily reshaping of the epithelial brush border against which the bacteria reside during the final 2 h prior to the daily expulsion (Wier et al., 2010).

As an environmentally transmitted symbiosis, the *Vibrio*-squid model has a number of valuable characteristics that have served it well as a study system for identifying molecular mechanisms. First, the binary system (two partners) is naturally reduced. Second, both partners can be raised separately and then introduced for experimentation. Third, *V. fischeri* is genetically tractable, and unbiased mutagenesis as well as precise genetic alterations can be introduced with relative ease. Fourth, the bacteria colonize the host light organ directly under the semi-transparent mantle and funnel; this permits imaging of the site of infection and direct analysis of bacterial behaviors and host responses. Fifth, synchronous colonization of hatchlings has permitted developmental staging of the colonization process. For most of the processes described below, many of these benefits were important in the advances described.

## From Pattern to Process in the *Vibrio*-Squid Symbiosis

**FIGURE 1:**
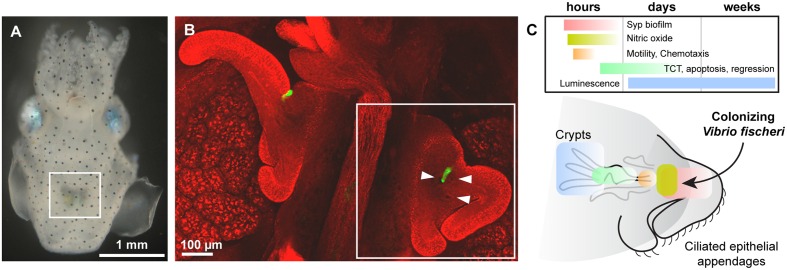
**(A)** Juvenile *Euprymna scolopes* hatchling, ventral view. White box highlights the ink sac and the light organ. **(B)** Confocal micrograph of the bilaterally symmetric light organ. Host tissue is counterstained in red and the colonizing bacteria are visible in green. Arrowheads point to the three pores on one side of the organ, into which *Vibrio fischeri* swim into the internal anatomy (ducts, antechamber, bottleneck, and crypts) of the organ. White box highlights one half of the organ, which is shown in cartoon view in the next panel. **(C)** Current state of knowledge about the temporal and spatial action of key processes discussed in this review, including Syp biofilm formation and aggregation (red), host nitric oxide (NO) production (yellow), bacterial motility and chemotaxis toward host chitin oligosaccharides (orange), symbiont TCT release (green), and luminescence (blue). In general the location of the colonizing bacteria are highlighted; e.g., for TCT release the bacteria colonize the crypts and release TCT (indicated), though the effect of this release on the host is apoptosis and regression of the ciliated epithelial appendages (not indicated in this representation). **(A,B)** Are adapted from Mandel et al. (2012).

In each section below, we highlight key discoveries in the *Vibrio*-squid symbiosis with a specific focus on how this model system has revealed molecular processes that underlie mutually beneficial phenotypes. **Figure 1** provides an overview to the juvenile light organ anatomy and the processes described in the article.

### Just the Two of Us

*Euprymna scolopes* squid light organs are colonized only by *V. fischeri*, and this exclusivity has guided substantial inquiry and discovery in the system. This pattern was first explored by McFall-Ngai and Ruby (1991) and extended in subsequent works (Ruby and Lee, 1998; Mandel et al., 2009). The ability to image the live animal during colonization enabled the discovery of *V. fischeri* aggregating in close proximity to the ciliated epithelial fields of the light organ (Nyholm et al., 2000). Nyholm discovered that a narrow distance between the green fluorescent protein-expressing bacteria and the squid epithelial tissue was the result of host-produced mucus, which included *N*-acetylneuraminic acid and *N*-acetylgalactosamine. Recent work has demonstrated that *V. fischeri* bind to cilia within this mucus field (Altura et al., 2013). Whereas many bacteria can bind in host mucus, only specific strains and species exhibit a competitive dominance over non-colonizing isolates, and only (some) *V. fischeri* strains proceed to fully initiate colonization (Nyholm et al., 2000; Nyholm and McFall-Ngai, 2003; Mandel et al., 2009).

Around this same time, the genetic basis for bacterial aggregation was being discovered and characterized in the laboratory of Karen Visick. A forward genetic screen for colonization factors first identified an orphan histidine kinase, RscS (regulator of symbiotic colonization-sensor), but without a phenotype or target it was difficult to know how this factor connected to the colonization process (Visick and Skoufos, 2001). The same screen identified an 18 gene locus that encoded regulatory proteins, glycosyltransferases, and other factors involved in exopolysaccharide production and export. Mutations in this region, the *syp* locus (symbiosis polysaccharide), conferred dramatic colonization defects in the animal as well as defects in biofilm formation in culture (Yip et al., 2005). A connection between these earlier studies was discovered when it was shown that RscS regulates expression of the *syp* locus (Yip et al., 2006). Overexpression of RscS provided a valuable tool in which bacterial colony formation took on a wrinkled or rugose colony morphology that is typical of biofilm formation (Yip et al., 2006). Phenotypes of *rscS* and *syp* alleles in colony-based biofilm assays map closely to their phenotypes during squid colonization, providing a valuable experimental tool for discovery and characterization of biofilm regulation. Further work has identified multiple layers of regulation, including a negative regulatory pathway that includes SypE and SypA, putative matrix proteins that integrate with the polysaccharide matrix, and a unique phosphorelay pathway (Visick, 2009; Morris and Visick, 2013; Norsworthy and Visick, 2015; Ray et al., 2015).

The genetic approaches described above (and in most studies in this review) were conducted in strain ES114, a squid isolate from Kaneohe Bay, Hawaii, that is used widely as a canonical squid symbiont. In addition to the biofilm regulatory pathway, a number of approaches including forward and reverse genetics studies had identified factors in strain ES114 that were important for squid colonization (Stabb and Visick, 2013). However, only some *V. fischeri* strains can colonize squid. Therefore, to examine the genetic basis for this host colonization specificity, Mandel et al. (2009) conducted a comparative genomic analysis of strains ES114 and MJ11, the latter being a fish symbiont that does not colonize squid robustly. The study determined that 91% of ES114 genes were almost identical between the squid and fish symbiont, but that approximately 400 genes in each strain were unique. Analysis of these factors revealed that the squid biofilm regulator, RscS, was encoded in the squid symbiont but not in the fish symbiont. The known RscS target genes, *sypA* through *sypR* were encoded in both genomes and fairly conserved (>85% amino acid identity). It was known previously that ES114 mutants that lacked RscS were unable to productively colonize the squid (Visick and Skoufos, 2001). Therefore, the study asked whether the absence of the regulator could explain the differential colonization phenotype. Introduction of RscS into strain MJ11 was sufficient to allow it to colonize the squid host. Phylogenetic analyses supported a model in which MJ11 was part of an ancestral group of *V. fischeri* that lacked *rscS*, and that this gene was acquired coincident with colonization of squid in the North Pacific Ocean (i.e., Japan and Hawaii; Mandel et al., 2009).

The idea that a single gene was sufficient to shift the animal hosts available to a bacterium was extreme but consistent with emerging literature that individual loci could impact microbe–host specificity. Work in entomopathogenic nematodes showed that symbiotic *Xenorhabdus nematophila* requires the three-gene *nilABC* locus for colonization, and that expression of these factors in a heterologous symbiont is sufficient to enable colonization of *Steinernema carpocapsae*, the worm host that otherwise is specific for *X. nematophila* (Cowles and Goodrich-Blair, 2008). Small genetic changes in *Yersinia pestis* have been key to its ability to colonize new niches, including single gene acquisitions and even inactivation of a gene already present (Sun et al., 2008, 2014; Zimbler et al., 2015). In the human gut microbiome there are examples in which single gene changes have been critical; e.g., in *Bacteroides fragilis*, polysaccharide A (PSA) confers a key immunomodulatory benefit that cannot be obtained from the other seven capsular polysaccharides produced (Mazmanian et al., 2008).

Studies on host colonization specificity in general, and biofilm formation in particular, have highlighted many of the strengths of the squid model. Imaging *in situ* was key to the initial discovery of the aggregates, forward genetics identified core exopolysaccharide synthetic and regulatory components, comparative genomics revealed the role of this pathway in the evolution and specificity of the association, and high-throughput genetic approaches are identifying additional levels of regulation. Additionally, this work highlights the value of model systems of beneficial bacteria, including *Vibrio* and *Xenorhabdus* models, to identify mechanistic details that resonate in beneficial and pathogenic colonization models.

### The Code Word is TCT

*E. scolopes* squid provide a particularly dramatic example of a role for bacteria influencing a specific host developmental process. Development of the host tissue proceeds on different trajectories depending on whether the specific symbiont *V. fischeri* is present. Only once the symbiont has colonized, the ciliated appendages of the host light organ undergo apoptosis, hemocyte infiltration, and tissue regression during the subsequent 5 days (McFall-Ngai and Ruby, 1991; Montgomery and McFall-Ngai, 1994; Koropatnick et al., 2004). The host morphogenesis is striking, with appendages that begin as outstretched mucus factories to recruit colonizing bacteria being reduced to small stumps (Montgomery and McFall-Ngai, 1994). As a result, it seems that initiation of the symbiosis is restricted to the first few days of the animal’s life while the appendages are present and secreting mucus.

How does the host know that the bacteria are inside to appropriately time the regression? It turns out that *V. fischeri* sheds envelope components that are received by receptors on the host. In particular, the bacterial peptidoglycan fragment, tracheal cytotoxin (TCT)-previously shown to induce a damaging apoptosis in ciliated epithelia upon release from *Bordetella pertussis*-was identified to perform a similar function in *V. fischeri*, but this time with a resulting beneficial outcome (Koropatnick et al., 2004). To recapitulate the apoptosis phenotype observed when intact *V. fischeri* are presented to the host, in the absence of the bacteria both the Lipid A portion of lipopolysaccharide (LPS) and TCT are required. The cell death from these compounds, in conjunction with hemocyte trafficking that is also induced from TCT, results in the regression phenotype. Previously these compounds had only pathogenic associations, but this work underscored a remarkable conservation to the cell biology of microbial–host interactions, emphasizing the context of the interaction to understand the fitness effects on the partners involved (Koropatnick et al., 2004).

Once the bacteria announce their arrival, how does the host speak back? In addition to regression of the appendages that recruit the bacteria, there are additional mechanisms by which the host receives and likely modulates the bacterial signal. Host nitric oxide (NO) production, described in more detail below, is diminished as a result of bacterial signaling (synergistically with LPS) (Altura et al., 2011). The host produces a peptidoglycan recognition protein, EsPGRP2, which is secreted into the bacterial-containing crypts and has the ability to degrade TCT (Troll et al., 2010). Additionally, there are data to suggest that host alkaline phosphatase, EsAP, modifies Lipid A after the initial signaling (Rader et al., 2012). In each case the host response is to diminish the potency of the bacterial products, but only after they have exerted their influence on host development.

This work in *V. fischeri* was influenced by studies in invertebrate systems that demonstrated host development in response to symbiont colonization and in vertebrates that showed general responses to consortia (reviewed in Montgomery and McFall-Ngai, 1994, and more recently in McFall-Ngai, 2014), and itself has influenced a field in which bacterial products play important roles in animal development. An early mammalian example by Hooper and Gordon demonstrated that in response to colonization by gut *Bacteroidetes* such as *Bacteroides thetaiotaomicron*, terminal tissue differentiation (e.g., fucosylation) is dependent on the presence of the symbiotic bacteria (Hooper and Gordon, 2001). There now exist many examples of bacteria directing specific host development. Recent exciting examples include *Algoriphagus machipongonensis* sulfonolipid signaling for multicellular rosette development in the choanoflagellate *Salpingoeca rosetta*, and *Pseudoalteromonas luteoviolacea* phage tail-like structures that stimulate tubeworm metamorphosis (Alegado et al., 2012; Shikuma et al., 2014).

### NO Way In

There is a long history of the study of NO in eukaryotes, and this small diffusible molecule has been implicated in many different cellular processes including signaling and innate immunity (Fang, 2004). Although the roles for NO in eukaryotic physiology and defense against pathogens were discovered many years ago, the study of this compound in the *Vibrio*-squid system and other symbioses (Damiani et al., 2016) has revealed that NO also influences the establishment and maintenance of mutualistic microbe–host relationships as both a signal and a specificity determinant (Wang and Ruby, 2011).

Davidson et al. (2004) first demonstrated that NO is produced in squid host tissue through the activity of nitric oxide synthase (NOS), and this activity was attenuated after successful colonization by *V. fischeri*. Using staining and immunocytochemistry, NOS and NO were found located in the epithelium of the light organ, as well as in vesicles within mucus shed from these cells. It is within this mucus that the bacterial cells aggregate prior to entering the light organ. Normally, *V. fischeri* aggregate in the mucus, colonize the host, and after successful colonization NOS activity and NO production are attenuated. Treatment of the animals with an NO-scavenging compound to diminish NO levels allowed large aggregates of non-symbiotic vibrios to form, but these bacteria did not successfully initiate colonization. (Davidson et al., 2004) The results suggested that NO acts as a specificity determinant, helping to limit aggregation of non-symbiotic vibrios and select for symbiotically competent *V. fischeri* from the mixed microbial population found in seawater.

If NO plays a role in specificity, then how do colonizing *V. fischeri* sense and respond to the host-produced NO to successfully establish the partnership? Using genetic approaches it was demonstrated that a strain lacking the NO-detoxifying enzyme flavohemoglobin (Hmp) displayed a colonization deficiency (Poole and Hughes, 2000; Wang et al., 2010b). Expression of *hmp* is regulated by the NO-responsive negative regulator NsrR (Rodionov et al., 2005; Tucker et al., 2010). However, NsrR is not the only important NO-sensing regulator in *V. fischeri*. H-NOX, a heme NO/oxygen-binding protein, also plays a role in symbiotically relevant NO-responsive regulation of genes in *V. fischeri* (Wang et al., 2010a). Although, H-NOX-like proteins are widely distributed in bacteria, this was the first report describing bacterial H-NOX function. Interestingly, it appears that one role for H-NOX in *V. fischeri* is to sense NO and correspondingly suppress bacterial hemin uptake during the early stages of host colonization. The authors predicted that early repression of iron uptake would protect the cells from the potentially harmful effects of Fenton chemistry when they are exposed to host-generated oxidants (Graf and Ruby, 2000; Davidson et al., 2004; Wang et al., 2010a). Consistent with this model, hemin uptake genes in *V. fischeri* were shown to be induced during the later stages of symbiotic colonization, and deletion of these genes negatively impacted colonization (Septer et al., 2011). Together, these studies support a model whereby host NO stimulates repression of hemin uptake genes; once bacterial colonization leads to an attenuation of host oxidant production, then hemin uptake genes are derepressed to support growth in the iron-limited light organ environment. Therefore, the ability to sense and detoxify NO is important for symbiotic specificity, and NO acts as a temporal signal to modulate bacterial gene expression and promote successful colonization.

Although, these studies have led to a better understanding of the role of a few key proteins and regulators in the response of *V. fischeri* to NO and the initial stages of the symbiosis, there is much yet to be learned about the global effects of NO on *V. fischeri* gene expression and metabolism, how this molecule acts as a specificity determinant, and whether there is a role for NO in the mature symbiosis. For example, the work of Wier et al. (2010) has suggested that NO may play a role in the daily symbiotic rhythm in the adult animal. Their data predicted that nitrate/nitrite respiration is used by the bacterial symbionts throughout the daylight hours. Similar to *Escherichia coli* (Vine and Cole, 2011), it is predicted that NO is produced by *V. fischeri* during respiration of nitrate/nitrite in laboratory culture. Endogenously produced NO could induce alternative respiratory pathways that likely influence the physiology and metabolism of the bacterium (Dunn et al., 2010). Together these separate lines of evidence suggest that NO may play a role beyond signaling and selection in the initiation of the symbiotic relationship. In the future it will be exciting to combine studies of NO and the bacterial NO response with the more recently developed ability to rear squid to adulthood (Koch et al., 2013; see section below on light production).

The value of further studies of NO in the *Vibrio*-squid system lie not only in providing important information about the role of this molecule in beneficial host–microbe interactions, but also for comparative studies to host–pathogen responses. Our current understanding supports a view that NO is being produced by the host and sensed by the bacteria in similar ways in many of the studied host–microbe interactions, whether the outcome of the relationship is beneficial or detrimental (Fang, 2004; Wang and Ruby, 2011). The prevalence of NO in host tissues colonized by bacteria suggests that a better understanding of the role of NO in symbiosis may have wide-reaching consequences for microbes at the interface of health and disease.

### Swimming against the Flow

In the mucus field that serves as the entry point for bacteria heading into the host, colonizing bacteria enter at one of three pores on either side of the bilaterally symmetrical light organ. Mucus is shed from the pores of the host at the same time that *V. fischeri* aggregates in that mucus. The bacteria proceed to migrate toward the pores, and each aggregate swims into a pore to colonize the ducts and crypts of the host. How do colonizing bacteria travel against this powerful flow? A key role for flagellar motility was identified over 20 years ago (Ruby and Asato, 1993). In that work Ruby and Asato confirmed that planktonic *V. fischeri* were motile due to a polar tuft of sheathed flagella. However, by 24 h-post-inoculation most cells in the light organ crypts were non-flagellated. Upon expulsion of bacteria from the host, the bacteria regrow their flagella in 45–60 min even in nutrient-deplete seawater (Ruby and Asato, 1993). Therefore, the bacterial life cycle alternates between a motile planktonic lifestyle and a non-flagellated crypt-colonized state.

Significant details have since been elucidated about the molecular mechanisms that control flagellar development in *V. fischeri*, which in turn has solidified the importance of swimming motility for squid colonization. Random transposon mutagenesis provided evidence that non-motile mutants could not colonize (Graf et al., 1994), and reverse genetics revealed that mutants defective for flagellar motility or chemotaxis did not establish productive colonization with the squid host (Millikan and Ruby, 2003, 2004; DeLoney-Marino and Visick, 2012). Together these studies established a model of a hierarchy of flagellar gene expression in *V. fischeri* controlled by the σ^54^-dependent regulator FlrA. There is evidence for regulation by quorum sensing and magnesium, and other sensory inputs are likely (O’Shea et al., 2005; Cao et al., 2012).

Bacterial flagellar motility often occurs in a directed fashion in which rotation of the flagellar bundle results in net movement toward preferred nutrient sources. Given the above information that chemotaxis was required for colonization, it seemed likely that the bacteria were swimming toward a host compound. The first evidence for chitin oligosaccharides as the specific attractant was obtained when addition of exogenous chitobiose, the *N*-acetylglucosamine dimer, blocked colonization, whereas the monomer did not have such an effect (Mandel et al., 2012). Given that *N*-acetylglucosamine is abundant on eukaryotic cell surfaces, yet chitin and its breakdown oligosaccharides are more specialized in their localization, it seemed possible that oligosaccharides may be a specific cue to direct entry into the host crypts. Mutants defective for chemotaxis remained at the outer face of the light organ pore, the same stage at which wild-type *V. fischeri* arrested their symbiotic development in the presence of added chitin oligosaccharides (Mandel et al., 2012). These results strongly suggested that host chitin served as a signal for the bacteria to enter the pore. Direct imaging revealed the presence of insoluble chitin bound to hemocytes within the host (Heath-Heckman and McFall-Ngai, 2011; Mandel et al., 2012), which may be released through the action of a host endochitinase (Kremer et al., 2013). Together, this illustrates a specific colonization checkpoint that is regulated by both host and symbiont factors.

Work on bacterial motility at the host interface has provided a valuable toolset to probe mechanisms of symbiosis and reveal novel signaling pathways. Many bacterial strains have dozens of genes that encode chemotactic sensory proteins, the methyl-accepting chemotaxis proteins (MCPs). The set of 43 MCPs in *V. fischeri* is typical in this regard, and despite difficulties in studying a large protein family, functions have now been assigned to three of these proteins. VfcA is the major amino acid chemoreceptor, and VfcB and VfcB2 are fatty acid chemoreceptors (Brennan et al., 2013; Nikolakakis et al., 2016). In addition to providing information directly about colonization, these tools provided insight into the role of LPS during colonization and for the evolution and the generation of torque at the flagellar motor (Post et al., 2012; Beeby et al., 2016). Furthermore, recent work suggests that the rotation of the flagellum-which is enclosed in an LPS sheath-stimulates outer membrane vesicle release and triggers the host immune response by promoting LPS release (Brennan et al., 2014; Aschtgen et al., 2016).

Satisfying answers to some of these questions are beginning to be revealed, including a role for cilia in modulating adhesion, as well as chemotaxis toward host-produced and host-cleaved chitin modulating a key developmental checkpoint. Still, important questions remain that suggest novel and interesting biology to be revealed through the symbiosis. Open questions include how bacteria transit through the mucus in a flagellar-independent manner; the molecular basis of chitin oligosaccharide sensing in the symbiont; and the processes that regulate the developmental switch between the aflagellate state in the host versus the swimming state in seawater.

### Light Up My Life

An important aspect to mutualistic symbioses is the selection of appropriate and cooperative partners. In both rhizobium-leguminous plant (Kiers et al., 2003) and *Vibrio*-squid symbioses the microbial partners provide costly services to their hosts (nitrogen fixation and light production, respectively). In theory, these relationships could be exploited by symbionts that are less cooperative (i.e., “cheaters”) (Ghoul et al., 2014). However, it is rare to find bacterial symbionts associated with the hosts that do not provide these services. Therefore, the *Vibrio*-squid mutualism provides an excellent model system for studying cooperative partner stability, and studies to date indicate that bacterial light production is required for bacterial cells to persist in the light organ.

*V. fischeri* is known to produce light in the squid host, and a key study demonstrated a role for luciferase, the enzyme that produces light, in bacterial symbiotic persistence (Visick et al., 2000). Mutants with defective luminescence structural genes or luminescence regulatory genes colonized juvenile squid to the same levels as wild-type in the first 24 h. However, by 48 h there was a 3- to 4-fold reduction in colonization by the dark mutants relative to wild-type controls. In squid co-colonized with both a luminescence mutant and wild-type, levels of the mutant strains similarly decreased, indicating that light-producing wild-type cells in the light organ could not complement the colonization defect of the light-deficient cells. These results suggested that the ability of individual bacteria to produce light was important for persistence in the light organ, and that somehow non-luminescent cells are selected against during development of the symbiosis.

Interestingly, the light-deficient strains have a specific effect on host development. Although colonization by a luminescence mutant still triggered apoptosis-related developmental changes in the ciliated surface of the light organ, colonization of the tissue by these strains no longer increased cell swelling of the epithelial cells lining the light organ crypt spaces. Therefore, light production appeared to play a specific role in host developmental pathways. Notably, this was the first report of *V. fischeri* genes required for induction of bacterial-triggered differentiation of host tissue (Visick et al., 2000).

It was later discovered that the antibiotic markers and method for constructing the early luminescence mutants (Visick et al., 2000) resulted in colonization attenuation and pleiotropic effects. Newly developed genetic tools were used to construct luminescence mutants that were not negatively affected in growth and colonization (Bose et al., 2008). Using these strains, the early results were confirmed demonstrating that the strain lacking the luminescence structural genes displayed a four-fold reduction in colonization as compared to wild-type at 48 h-post-inoculation.

Previous studies suggested that maintenance of the symbiosis over the life of the animal requires a maturation process of several weeks (Montgomery and McFall-Ngai, 1998), leaving the question of how production of light influences symbiosis maturation beyond 72 h. A major breakthrough for the field came with the development of protocols for simplified rearing of newly hatched juvenile squid through and beyond the maturation process. These methods allowed investigation of how bacterial-produced light affects the development of the symbiosis over 4 weeks (Koch et al., 2013). In these studies, the levels of the luminescence-deficient mutant associated with the squid light organ continued to diminish over time, to the minimum level of detection after 28 days. Similar results were observed in squid colonized with mixed inocula containing both wild-type and the luminescence mutant, where after 15 days the mutant was barely detected. Therefore, the persistence defect observed during early colonization becomes more pronounced as the symbiosis matures, with eventual loss (or near loss) of non-luminescent strains in a matter of weeks.

Luminescence regulation is one of the hallmarks of the *V. fischeri*-squid symbiosis and has been studied intensively, yet there are still exciting open questions. First, how are the dark mutants removed from the population even in the midst of neighboring bright populations? A clue comes from studies testing the influence of a previous colonization event on recolonization (Koch et al., 2013). Juvenile animals were colonized with either wild-type or a luminescence mutant. After 1–5 days, the animals were treated with antibiotics to clear bacteria from the light organ and then exposed again to wild-type *V. fischeri* to test whether light production is a “signal” to the host that influences symbiotic maturation. Animals treated with antibiotics after 1 day were readily recolonized, regardless of the strain that initially colonized. However, after 5 days, wild-type *V. fischeri* induced a refractory state in the animal that prevented recolonization. In contrast, in animals initially colonized by a luminescence mutant, greater than 80% of the animals were recolonized by wild-type. These results support the idea that the host is detecting light production by bacterial cells and/or is altering physiological conditions to sanction the non-luminescent strains. In addition, the host apparently is able to “eject” an inappropriate light deficient strain-directly or indirectly-while allowing future recolonization by a symbiotically appropriate light-producing strain. The exact mechanisms by which the detection, sanctioning, and/or ejection occurs remain to be described. The host does have the capacity to detect light but it is unknown whether this capacity is connected to symbiont selection (Tong et al., 2009).

A second interesting question relates to how bacterial light production is matched to the moonlight in such an exquisite fashion. The squid contains elaborate tissues to physically reflect and modulate bacterial light production (Crookes et al., 2004). This physical response could be triggered through the activity of products of host cryptochrome and eye-specification genes; the expression of these genes appears to be influenced by the light produced by *V. fischeri* (Heath-Heckman et al., 2013; Peyer et al., 2014). The physical reflection and modulation of bacterial luminescence is also coordinated with a molecular signaling response. For example, host epithelial cells swell in response to light-producing strains but not dark mutants (Visick et al., 2000). This swelling could release chemical cues into the light organ environment. Recent evidence indicates that bacterial luminescence in the light organ is controlled not only through quorum sensing, but also through response to environmental signaling (Septer and Stabb, 2012). These results suggest there is complex chemical and physical control of light production in the symbiosis. Bacterial luminescence is a particularly intriguing and engaging aspect of the *Vibrio*-squid symbiosis, and it is clear that there are abundant questions remaining to be addressed as to how the interaction with the host and the environment lead to specific phenotypic output in the host.

### Nice To Meet You… Now What Is It You Do?

The *Vibrio*-squid symbiosis has provided a useful framework for identifying the function of bacterial genes and studying novel genes *in vivo*. Due to the wealth of genetic tools that have been developed for *V. fischeri* and the ability to access the host interface with direct imaging, it is possible to test the effects of gene loss in the real-world environment of the host. Two examples discussed below are using the *Vibrio*-squid system to broaden understanding of gene function for alternative oxidase (AOX) and for discovering the role of the biofilm inhibitor BinK.

Alternative oxidase is a terminal respiratory oxidase that is ubiquitous in plants, and is unusual because its activity is not directly linked to generation of the proton motive force (Vanlerberghe and McIntosh, 1997). The study of the function of AOX in plants is an active area of research, and AOX function has been linked to both abiotic and biotic stress responses (Vanlerberghe, 2013). Only with the explosion of genome and metagenome sequencing was it discovered that certain bacterial genomes also encode this protein (Stenmark and Nordlund, 2003), and that *aox*-like genes are abundant in metagenomic sequences from ocean surface waters (McDonald and Vanlerberghe, 2005). However, early progress toward understanding the physiological benefit of AOX function in bacteria was limited by the lack of genetic tools for many of the AOX-encoding organisms. A path to revealing a functional role for AOX came with the discovery that the genome of *V. fischeri* strain ES114 encoded AOX (Ruby et al., 2005). A transcriptomic analysis of the *V. fischeri* response to NO revealed that NO induces expression of *aox* (Wang et al., 2010a). The connection to NO was further clarified through characterization of the role of the NO-responsive negative regulator NsrR in regulation of *aox* expression, and identification of the ability of *V. fischeri* AOX to function as an NO-resistant oxidase (Dunn et al., 2010). Despite the known connections between *aox* and NO, and between NO and the early stages of host colonization, no discernible phenotypic difference between the *aox* mutant and wild-type in early colonization of the squid host has been observed. Although, there is the possibility that AOX does not play a role in bacterial physiology during host colonization, an alternative explanation is that the benefit of AOX expression during colonization does not result in a phenotype dramatic enough to be detected in the short time frame of the experiments (1–3 days). Experiments to test this possibility are in progress and would be consistent with studies above described for luminescence mutants in which colonization phenotypes change over the course of symbiosis and effects are magnified over a multi-week time course.

Studying AOX regulation and function in *V. fischeri* as a model organism will provide a framework for understanding how bacteria in ocean surface waters utilize this respiratory pathway in growth and survival. Work is underway to clarify the physiological benefit of AOX function in *V. fischeri* and other *aox*-containing bacteria, with the ultimate goal of better understanding how bacteria cope with changing conditions in the environment. Studying AOX in the context of the symbiosis has provided insight into the expression and function of this interesting protein, and provides a framework for broad studies of how AOX function influences bacterial physiology in the environment.

Study of AOX followed a reverse-genetic approach, starting with identification of an interesting gene through genome sequencing, and through directed experimental approaches leading to a better understanding of gene function. However, in many cases forward genetic approaches have identified genes whose products are relevant for a specific colonization process. An excellent example is *binK*, which encodes a histidine kinase. Above we described a key role for biofilm formation in the colonization process as regulated by RscS and Syp. In a recent global genetic screen for mutants with an advantage in squid colonization, *binK* was identified as a locus that when disrupted resulted in substantially better colonization of the *V. fischeri* strain (Brooks and Mandel, 2016). Typical means to predict protein function (e.g., homology, neighboring genes) were not helpful, so phenotypes of cells lacking *binK* were examined in culture and in the host and revealed a substantial increase in symbiotic biofilm formation. BinK (biofilm inhibitor kinase) is therefore a negative regulator of biofilm formation and an additional membrane-bound histidine kinase that is critical for proper regulation of the Syp biofilm.

In the case of both AOX and BinK, the depth of the *V. fischeri*-squid system has provided a means to assign function to novel and poorly understood proteins. A striking number of genes are poorly understood in bacterial genomes, exemplified by the 149 (32%) of the minimal 473 genes in the JCVI-syn3.0 genome with functions that remain to be discovered (Hutchison et al., 2016). The ability to study biological function in the context of the host thus provides a useful lens through which to identify and characterize genes and their products.

## Conclusion

The *Vibrio*-squid system has proven to be a valuable study system for identifying principles of microbe–host interactions, continues to serve as a fertile field for discovery, and provides a useful road map for moving from patterns of intriguing phenotypes to discerning the molecular communication between microbe and host that is responsible for those patterns. By integrating approaches in genetics, genomics, molecular biology, imaging, physiology, evolutionary biology, and cell biology, each of the topic areas highlights an integrated and mechanistic view of how symbiotic partners functionally communicate in a model microbiome. In this manner, the *Vibrio*-squid system provides a durable example for how to move from fascinating observations to molecular understanding of the processes by which very different organisms communicate and establish a productive partnership.

## Author Contributions

All authors listed, have made substantial, direct and intellectual contribution to the work, and approved it for publication.

## Conflict of Interest Statement

The authors declare that the research was conducted in the absence of any commercial or financial relationships that could be construed as a potential conflict of interest.
